# Protein Corona Fingerprints of Liposomes: New Opportunities for Targeted Drug Delivery and Early Detection in Pancreatic Cancer

**DOI:** 10.3390/pharmaceutics11010031

**Published:** 2019-01-15

**Authors:** Sara Palchetti, Damiano Caputo, Luca Digiacomo, Anna Laura Capriotti, Roberto Coppola, Daniela Pozzi, Giulio Caracciolo

**Affiliations:** 1 Department of Molecular Medicine, Sapienza University of Rome, Viale Regina Elena 291, 00161 Rome, Italy; sara.palchetti@uniroma1.it (S.P.); luca.digiacomo@uniroma1.it (L.D.); 2 Department of General Surgery, University Campus-Biomedico di Roma, Via Alvaro del Portillo 200, 00128 Rome, Italy; d.caputo@unicampus.it (D.C.); r.coppola@unicampus.it (R.C.); 3 Department of Chemistry, Sapienza University of Rome, P.le Aldo Moro 5, 00185 Rome, Italy; annalaura.capriotti@uniroma1.it; 4 Istituti Fisioterapici Ospitalieri, Istituto Regina Elena, Via Elio Chianesi 53, 00144 Rome, Italy

**Keywords:** pancreatic ductal adenocarcinoma, liposomes, protein corona

## Abstract

Pancreatic ductal adenocarcinoma (PDAC) is the fourth cause of cancer-related mortality in the Western world and is envisaged to become the second cause by 2030. Although our knowledge about the molecular biology of PDAC is continuously increasing, this progress has not been translated into better patients’ outcome. Liposomes have been used to circumvent concerns associated with the low efficiency of anticancer drugs such as severe side effects and damage of healthy tissues, but they have not resulted in improved efficacy as yet. Recently, the concept is emerging that the limited success of liposomal drugs in clinical practice is due to our poor knowledge of the nano–bio interactions experienced by liposomes in vivo. After systemic administration, lipid vesicles are covered by plasma proteins forming a biomolecular coating, referred to as the protein corona (PC). Recent studies have clarified that just a minor fraction of the hundreds of bound plasma proteins, referred to as “PC fingerprints” (PCFs), enhance liposome association with cancer cells, triggering efficient particle internalization. In this study, we synthesized a library of 10 liposomal formulations with systematic changes in lipid composition and exposed them to human plasma (HP). Size, zeta-potential, and corona composition of the resulting liposome–protein complexes were thoroughly characterized by dynamic light scattering (DLS), micro-electrophoresis, and nano-liquid chromatography tandem mass spectrometry (nano-LC MS/MS). According to the recent literature, enrichment in PCFs was used to predict the targeting ability of synthesized liposomal formulations. Here we show that the predicted targeting capability of liposome–protein complexes clearly correlate with cellular uptake in pancreatic adenocarcinoma (PANC-1) and insulinoma (INS-1) cells as quantified by flow-assisted cell sorting (FACS). Of note, cellular uptake of the liposomal formulation with the highest abundance of PCFs was much larger than that of Onivyde^®^, an Irinotecan liposomal drug approved by the Food and Drug Administration in 2015 for the treatment of metastatic PDAC. Given the urgent need of efficient nanocarriers for the treatment of PDAC, we envision that our results will pave the way for the development of more efficient PC-based targeted nanomaterials. Here we also show that some BCs are enriched with plasma proteins that are associated with the onset and progression of PDAC (e.g., sex hormone-binding globulin, Ficolin-3, plasma protease C1 inhibitor, etc.). This could open the intriguing possibility to identify novel biomarkers.

## 1. Introduction

With a one-year survival rate of 12% that declines to 1% at five years, pancreatic ductal adenocarcinoma (PDAC) is one of the most lethal tumors worldwide [1]. It is currently the fourth leading cause of cancer-associated mortality and it is predicted to become the second leading cause in the next decade in Western countries [2]. When PDAC is diagnosed, surgery remains the only treatment chance, while chemotherapeutic agents are often inefficacious. To tackle this issue, numerous drugs have been tested. Gemcitabine (GEM) was the first drug to be approved for pancreatic cancer, but it is currently used only as a palliative agent [3]. Cisplatin and 5-Fluorouracile can extend life for a few months but both have collateral toxic properties [4]. Irinotecan is an antitumor drug belonging to the camptothecin family that targets DNA topoisomerase-1, a nuclear enzyme able to prevent torsional stress during DNA replication and transcription [5]. Nanotechnology has recently gained attention for its ability to treat numerous tumors, with nanocarriers being used to circumvent the problems associated with anticancer drugs, including high toxicity and irreversible damage of normal cells [6]. Recently, Onivyde^®^, an Irinotecan liposomal formulation, has been approved by the Food and Drug Administration (FDA) for the treatment of metastatic pancreatic cancer resistant to gemcitabine chemotherapy [7]. As a matter of fact, encapsulated liposomal drugs exhibit better pharmacokinetics and therapeutic index, as well as reduce the collateral toxic effects of free drugs. However, the adsorption of plasma opsonins (e.g., complement proteins, immunoglobulins, etc.) to the liposomal surface results in the clearance of liposomes from the blood circulation [8]. For a couple of decades, researchers have tried to prevent protein binding by grafting polymers to the liposome surface and, in this regard, polyethylene glycol (PEG) has been the gold standard for stealth polymers in drug delivery [9]. Conjugating PEG terminals to tissue-recognition ligands (e.g., peptides, antibodies, etc.) has long been supposed to provide such “long-circulating” liposomes with selective targeting ability [10]. However, recent findings have demonstrated that grafting polymers to a liposome surface can only reduce protein binding, but cannot fully prevent it [11]. Moreover, Schöttler et al. showed that PEG promotes the recruitment of specific plasma proteins [12], thus contributing to explain the accelerated blood clearance (“ABC phenomenon”) of PEGylated nanosystems [13]. The main implication is that active targeting usually fails in vivo with the result that no targeted liposomal drug has been approved so far. While protein binding to a liposome surface is a well-established paradigm in drug delivery [14,15,16], the emerging field of nano–bio interactions between nanosized objects and biological systems is putting earlier findings in context, providing the liposome field with new perspectives [17,18,19,20].

**Figure 1:**
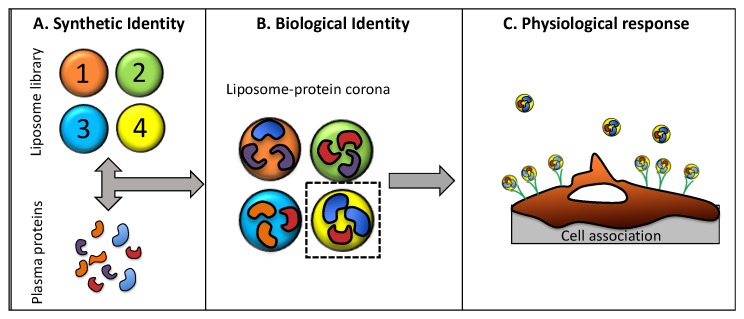
Schematic illustrating the protein corona fingerprinting strategy. (**A**) A library of liposomes is mixed with plasma proteins; (**B**) plasma proteins adsorb to the particle surface, forming liposome–protein complexes that are ranked for enrichment in protein corona ‘fingerprints’, i.e., plasma proteins that promote association with cancer cells; (**C**) selected formulations are incubated with cells in culture and cell association is measured by flow-assisted flow cytometry.

When liposomes are introduced into a biological fluid, they are covered by a dynamic layer of biomolecules, in particular proteins, forming the so-called “protein corona” (PC) [21,22]. This complex interface is formed in seconds and, over time, it changes prevalently in the amount of bound protein and slightly in protein composition [23]. With respect to other kinds of nanoparticles, the liposome–PC evolves significantly during the first hour of exposure to biological fluids [24] and is the reason why exposure time is typically fixed at 1 h [24,25,26]. As a consequence of PC formation, liposomes lose their synthetic identity and attain a new one that is usually referred to as their “biological identity”. It is this newly acquired biological identity that controls undesirable side effects of liposomal drug delivery, such as off-target interactions, toxicity, size-dependent particle recognition by immune cells [27], and clearance from the bloodstream [28,29]. On the other side, it is increasingly accepted that even particle accumulation at the target site is controlled by the biological identity acquired in biological environments [17]. For instance, non-specific interactions between liposomes and target cells are controlled by physical-chemical properties (i.e., size and zeta-potential) of liposome–protein complexes and not by those of pristine liposomes. Moreover, the PC may act as an endogenous trigger, promoting association with receptors of target cells and leading to efficient internalization. In a couple of recent investigations [26,30], we demonstrated that liposomes possessing specific size and zeta-potential are efficiently internalized within cancer cells [26,30]. Moreover, a minor fraction of identified “corona proteins” (typically 1–2%), referred to as “protein corona fingerprints” (PCFs), promote favorable cellular association. Globally, liposome physical-chemical properties, PC composition, and cellular uptake can be combined in a general strategy to predict the interaction of liposomes with cancer cells (Figure 1).

This work was therefore aimed at exploiting the liposome–PC to target human pancreatic carcinoma (PANC-1) cells. To this end, a library of 10 liposomal formulations was synthesized and liposome–protein complexes were thoroughly characterized by dynamic light scattering (DLS), micro-electrophoresis (ME), and nano-liquid chromatography tandem mass spectrometry (nano-LC MS/MS). Next, liposomes were screened for their particle properties and corona composition. Of note, cellular uptake by PANC-1 cells was found to correlate with physical-chemical properties of liposome–protein complexes and enrichment in PCFs. A second aim of the study was the complete identification of the protein patterns adsorbed to synthesized liposomes. Indeed, the recently introduced concept of the “disease-specific PC” [31] states that the PC composition is affected by changes in human proteome as those induced by numerous diseases such as cancer. Thus, identifying proteins that are related to pancreatic tumor onset and progression could pave the way to identify cancer in the early stages by differential analysis of the PC.

## 2. Materials and Methods

### 2.1. Liposomes Preparation

Cationic lipids 1,2-dioleoyl-3-trimethylammonium-propane (DOTAP) and (3β-[*N*-(*N*′,*N*′-dimethylaminoethane)carbamoyl])cholesterol; neutral lipids dioleoylphosphatidylethanolamine (DOPE), 1,2-dipalmitoyl-*sn*-glycero-3-phosphocholine (DPPC), and 1,2-diarachidoyl-*sn*-glycero-3-phosphocholine (20:0 PC); the zwitterionic lipid dioleoylphosphocholine (DOPC); and the anionic lipid 1,2-dioleoyl-*sn*-glycero-3-phospho-(1′-rac-glycerol) (DOPG) were purchased from Avanti Polar Lipids (Alabaster, AL, USA), while sphingosine and cholesterol were from Sigma-Aldrich (St. Louis, MO, USA). All lipids were used without further refinement and were prepared at desired molar ratios. Each lipid was dissolved in chloroform and the solvent was evaporated under a vacuum for at least 2 h. Lipid films were hydrated in ultrapure water to obtain a final lipid concentration of 1 mg/mL. The obtained liposome solutions were extruded 20 times through a 0.1-μm polycarbonate carbonate filter with the Avanti Mini-Extruder (Avanti Polar Lipids, Alabaster, AL, USA). Liposomes were incubated with human plasma (HP) (1:1 *v*/*v*) for 1 h at 37 °C. Incubation time was chosen according to previous findings as it represents a typical plateau of the temporal evolution of the liposome–PC [24].

### 2.2. Size and Zeta-Potential Experiments

For size and zeta-potential experiments, bare liposomes and liposome–HP complexes were diluted 1:100 with ddH_2_O. All the measurements were performed using a Zetasizer Nano ZS90 (Malvern, UK) at room temperature. Experiments were made in triplicate and the results are given as means ± standard deviation.

### 2.3. Proteomics Experiments

Lipid films were hydrated with a dissolving buffer (Tris-HCl, pH 7.4, 10 mmol L^−1^; NaCl, 150 mmol L^−1^; EDTA, 1 mmol L^−1^). The obtained solutions were extruded 20 times through a 0.1-μm polycarbonate carbonate filter with the Avanti Mini-Extruder (Avanti Polar Lipids, Alabaster, AL, USA) and stored at 4 °C until use. Liposomes were incubated with HP (1:1 *v*/*v*) and then incubated at 37 °C for 1 h. After incubation, samples were centrifuged for 15 min at 14,000 rpm. Pellet was robustly washed with phosphate-buffered saline (PBS) and resuspended. This procedure was repeated three times to wash the sample and remove loosely bound proteins. Protein denaturation, digestion, and desalting were carried out by a robust methodology that is commonly used to separate liposome−PC complexes from unbound and loosely bound proteins [11]. In brief, samples were lyophilized by a Speed-Vac apparatus (mod. SC 250 Express; Thermo Savant, Holbrook, NY, USA). Samples were reconstituted with 0.1% HCOOH solution (final concentration 0.32 mg/mL) and stored at −80 °C until LC MS/MS was carried out. Tryptic peptides were investigated by a nano-LC system (Dionex Ultimate 3000, Sunnyvale, CA, USA) connected to a hybrid mass spectrometer (Thermo Fisher Scientific, Bremen, Germany), equipped with a nanoelectrospray ion source. Xcalibur (v.2.07, Thermo Fisher Scientific) raw data files were submitted to Proteome Discover (1.2 version, Thermo Scientific) for a database search using Mascot (version 2.3.2 Matrix Science). Data was searched against the SwissProt database (v 57.15, 20,266 sequences) using the decoy search option of Mascot and protein quantification was made by Scaffold software. For each identified protein, the mean value of the normalized spectral countings (NSCs) was normalized to the protein molecular weight (MWNSC) to obtain the relative protein abundance (RPA) [32]. For each identified protein, the reported RPA is the mean of three independent replicates ± standard deviation.

### 2.4. Cell Culture

Human pancreatic carcinoma cell line (PANC-1) was purchased from Sigma-Aldrich (St. Louis, MO, USA) and maintained in DMEM medium. Rat insulinoma cell line (INS-1) was purchased from Thermo Fisher (Waltham, MA, USA) and was maintained in RPMI. Both mediums were supplemented with 2 mM L-glutamine, 100 IU/mL penicillin-streptomycin, 1 mM sodium pyruvate, 10 mM Hepes, 1.5 mg/L sodium bicarbonate, and 10% fetal bovine serum. Cell lines were cultured at 37 °C in a humidified atmosphere with 5% CO_2_.

### 2.5. Flow-Assisted Cell Sorting Experiments

For cellular uptake experiments, Lip-1 and Lip-5 liposomes were synthesized using Texas Red^®^ 1,2-dihexadecanoyl-*sn*-glycero-3-phosphoethanolamine, triethylammonium salt (TX-DHPE) (Thermo Fisher, Waltham, MA, USA). Onyvide-like liposomes were prepared using DSPC, Chol, MPEG-2000-DSPE, and TX-DHPE at the molar ratios 215:143:1:1. Cells were seeded on 12-well plates (150,000 cells/well) in complete medium and, after 2 h, cells were treated with liposomes incubated with human plasma for 1 h using Optimem medium. After 3 h, cells were detached with trypsine/EDTA, washed two times with cold PBS, and run on a BD LSR Fortessa^TM^ (BD Bioscience, San Jose, CA, USA).

## 3. Results and Discussion

First, we synthesized a combinatorial library of 10 liposomal formulations. According to previous findings [33,34], liposomes were prepared by mixing cholesterol, DC-Chol, DOPC, DOPE, DOTAP, DPPC, PC (20:0), and sphingosine in specific molar ratios (Table 1).

**Figure 2:**
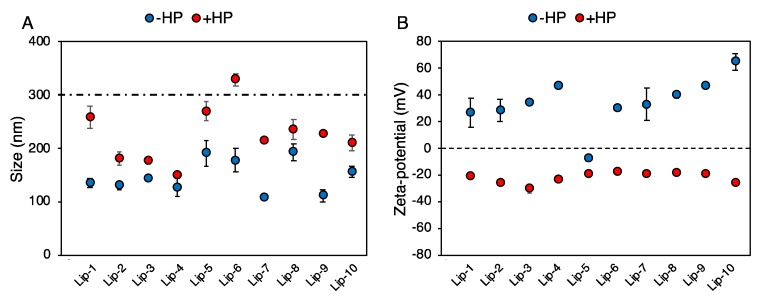
(**A**) Hydrodynamic diameter of liposomes before (blue points, “− human plasma (HP)”) and after (red points, “+ HP”) 1-h incubation with human plasma (HP) of pancreatic cancer patients. Values are means ± standard deviation from three independent experiments. The dashed line indicates a typical size threshold for particle removal from bloodstream by macrophages. (**B**) Zeta-potential of liposomes before (blue points, “− HP”) and after (red points, “+ HP”) 1-h incubation with human plasma (HP) of pancreatic cancer patients.

Next, the synthetic identity of liposomes (i.e., size, zeta-potential, and aggregation state post-synthesis) was characterized by DLS and ME (Figure 2). Pristine vesicles were small in size with a hydrodynamic diameter (D_H_) ranging from ~100 nm to 150 nm (Figure 2A, blue points). Moreover, the polydispersity index (PDI) indicated that all liposomal formulations were monodisperse (Table 2).

Zeta-potential of liposomes varied between ~0 mV (Lip-5) and ~65 mV (Lip-10) depending on liposomal lipid composition (Figure 2B, blue points). One-hour exposure to HP lead to the formation of liposome–protein complexes that were characterized in terms of size, zeta-potential, and homogeneity of dispersion. The size of liposome–protein complexes (Figure 2A, red points) was larger than that of pristine vesicles and varied appreciably among formulations. This is in full agreement with previous findings showing that lipid composition plays a key role in protein binding to a lipid surface [35]. According to the literature [22], a size increase of a few nanometers is likely due to the formation of a PC on the liposome surface, while an enlargement of a few tenths of a nanometer reflects the clustering of single liposomes coated by plasma proteins [25,32].

Liposome aggregation is confirmed by an increase in PDI values (Table 2). On the other side, “normalization” in zeta-potential around −20 mV (Figure 2B, red points), regardless of pristine surface charge, has been reported for many classes of nanomaterials and is caused by the fact that most plasma proteins have a negative charge at physiological pH. Besides the size and zeta-potential of liposome–protein complexes, the biological identity of liposomes is also controlled by the composition of the PC. When in the blood, the liposome–PC could hamper the ability of the pristine vesicle to bind to target receptors [36] and may induce activation of the immune system, leading to particle clearance [21,37].

On the other hand, molecular recognition between endogenous plasma proteins (i.e., recruited from the blood) and cancer cell receptors [38] could promote selective accumulation at the tumor site. A crucial step towards the exploitation of the PC for targeted drug delivery is therefore the identification and quantification of corona proteins. Bradford assay results showed that the amount of bound protein is dependent on both the zeta-potential and lipid composition (Table 3).

Generally, it was observed that cationic liposomes adsorb more proteins than neutrally charged vesicles. Likewise, nano-LC MS/MS showed that the liposome–PCs were highly complex entities containing between 140 and 222 proteins (Table 4).

**Figure 3:**
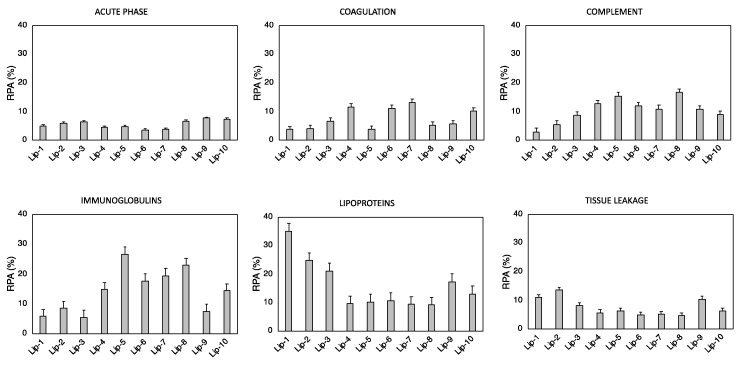
Bioinformatic classification of proteins identified in the corona of Lip-1–Lip-10 after 1-h exposure to HP. The relative protein abundances (RPAs) of total proteins are shown.

The identified number of proteins in Table 4 is larger than that accommodated on the liposome surface. This apparent discrepancy was clarified by recent models that describe the corona as a coating made of several layers held together by protein–protein interactions [39,40]. To facilitate their rational identification, corona proteins were grouped according to physiological functions of the blood system. The relative protein abundance (RPA) of biologically relevant proteins such as complement proteins, coagulation proteins, immunoglobulins, acute phase proteins, tissue leakage, and lipoproteins are displayed in Figure 3.

Our findings confirmed that each liposome exhibits a specific protein pattern dictated by its specific lipid composition. Over the last decade, numerous studies have tried to relate the cellular uptake of nanoparticle–protein complexes to PC composition by an oversimplified picture of particle–cell interaction; the more abundant a corona protein, the more probable the molecular recognition by cell receptors and, in turn, the more significant its role in promoting nanoparticle–cell association. However, to understand the link between a nanoparticle–corona complex and specific uptake pathways, mapping the exact location of protein binding sites is a necessary step [41,42]. To date, mapping protein epitopes at the liposome surface is challenging. To overcome this issue, computational methods such as quantitative structure–activity relation (QSAR) allow the identification of the most appropriate set of descriptors to predict the interactions between liposomes and cells at the nano–bio interface [43,44]. Correlations between the RPA of individual proteins and cellular uptake allowed us to identify eight “protein fingerprints” (Vitronectin, APOA1, APOA2, APOB, APOC2, Ig heavy chain V-III region BRO, vitamin K-dependent protein, and Integrin beta3) that promote the association of liposomes with cancer cells [26,30]. Among PCFs, a key role is played by Vitronectin, a glycoprotein of the hemopexin family containing an RGD motif (Arg-Gly-Asp) in the Somatomedin B domain (20−63 region) that is specifically recognized by α_ν_β_3_ integrins [38]. This class of integrins is overexpressed on many solid tumors and in tumor neovasculature [45,46]. This could be extremely relevant in pancreatic cancer, where roughly half of patients show elevated expression of αvβ3, and this is positively correlated with lymph node metastasis [47]. Most chemotherapeutics given in the clinic today damage healthy tissues, leading to unwanted side effects. According to our present understanding, this could likely be related to off-target interactions between corona proteins and cell receptors of healthy cells. This means that receptors targeted by corona proteins should be overexpressed in cancer but not in normal cells. In this regard, it is known that α_v_β_3_ integrin is expressed by normal (i.e., not-cancer) cells in a latent state characterized by its inability to stimulate cell adhesion to extra-cellular matrix ligands [48]. We also observed that, of eight PCFs, four are Apolipoproteins. It is well known that Apolipoproteins bind certain receptors such as scavenger receptor class B, type I (SR-BI), and low-density lipoprotein receptors (LDLR) that are overexpressed in several diseases. Recent studies showed that SR-BI and LDLR are overexpressed in pancreatic cancer, thus representing good targets of Apolipoprotein-enriched coronas [49,50]. Liposomal formulations were ranked for their PC-based targeting ability by calculating the total abundance of PCFs [51].

**Figure 4:**
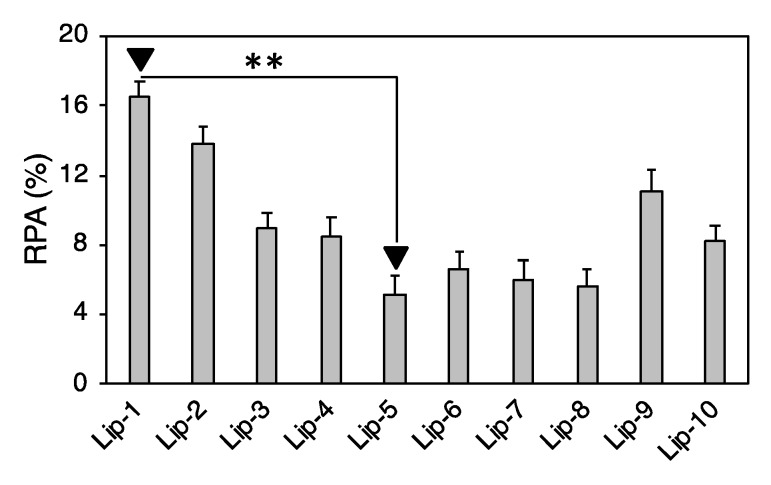
Relative protein abundance of biomolecular corona fingerprints. Lip-1 and Lip-5 were the liposomal formulations with the highest and lowest enrichment in PCFs (Vitronectin, APOA1, APOA2, APOB, APOC2, Ig heavy chain V-III region BRO, vitamin K-dependent protein, and Integrin beta3). Significance was statistically evaluated by Student’s *t*-test (** *p* < 0.05).

**Figure 5:**
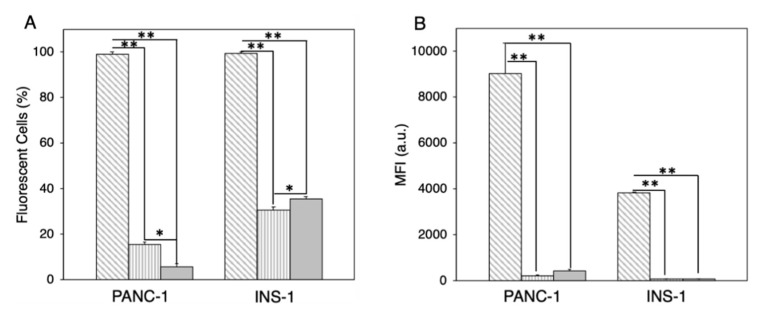
(**A**) Cellular uptake of Lip-1 (grey diagonal hatched lines), Lip-5 (grey vertical hatched lines), and Onivyde^®^ (grey bar) in PANC-1 and INS-1 cells after 1-h incubation with human plasma (HP). (**B**) Mean fluorescence intensity of Lip-1 (grey diagonal hatched lines), Lip-5 (grey vertical hatched lines), and Onivyde^®^ (grey bar) in PANC-1 and INS-1 cells after 1-h incubation with human plasma (HP). Statistical significance was evaluated using Student’s *t*-test: * *p* < 0.01; ** *p* < 0.005 (no asterisk means lack of significance).

According to Figure 4, Lip-1 was identified as the most promising formulation to promote cellular association within PANC-1 and INS-1 cells. On the other side, Lip-5, being highly defective in PCFs, was expected to promote low cellular internalization. To support our conclusions, we treated PANC-1 cells with fluorescently labeled Lip-1- and Lip-5-protein complexes. Onivyde^®^, the liposomal formulation approved by the FDA for the treatment of metastatic pancreatic cancer [7,52], was used as a control. In order to obtain a quantitative view on this process, we performed FACS analysis. Figure 5A shows that about 95% of PANC-1 and INS-1 cells treated with Lip-1-protein complexes were fluorescence-positive. On the other hand, Lip-5-protein complexes were poorly internalized by PANC-1 cells, as demonstrated by the fact that less than 20% were positive for the fluorescence signal. This percentage was slightly higher in INS-1 cells (<30%). FACS results also show that the internalization of Onivyde^®^ in both PANC-1 and INS-1 cells is extremely low, with only 10% and 20% of fluorescence-positive cells, respectively. The mean fluorescence intensity reported in Figure 5B shows the same trends as those observed for cellular uptake.

Lastly, our MS/MS results indicate that the composition of the PC (in terms of types and amounts of the constituent proteins) depends strongly on the physical-chemical properties of the liposomes. In particular, we observed that the coronas of Lip-1 and Lip-5 were particularly enriched with plasma proteins and were associated with the onset and progression of pancreatic cancer (e.g., sex hormone-binding globulin, Ficolin-3, plasma protease C1 inhibitor, etc.). Recently, some authors introduced the concept of the disease-specific PC [31], wherein alterations in human proteome of patients with various diseases produce appreciable changes in the PC protein pattern. Consequently, we envision that the manipulation of liposome surface chemistry can dictate the selective binding of plasma proteins with the possibility of identifying cancer at the early stages.

## 4. Conclusions

In conclusion, we have synthesized a library of 10 liposomal formulations that exhibit peculiar biological identities when exposed to HP. We found that the formulation exhibiting the highest levels of targeting fingerprints also had major cellular uptake in PANC-1 and INS-1 cells. Our results indicate that the exploitation of PCs could be a valuable means to develop targeted nanomedicine for PDAC treatment. Moreover, we found that the PCs of some liposome formulations were enriched with plasma proteins that are related to PDAC onset and progression. This possibility could pave the way for the identification of novel biomarkers and will be explored in future investigations.

## Figures and Tables

**Table 1:** The molar ratios of lipids used for synthesized a library of 10 liposomal formulations.

Samples	CHOLESTEROL	DC-CHOL	DOPC	DOPE	DOTAP	DPPC	PC (20:0)	SPHINGOSINE
Lip-1	0	0	0	0.5	0.5	0	0	0
Lip-2	0	0.5	0	0.5	0	0	0	0
Lip-3	0	0.25	0.25	0.25	0.25	0	0	0
Lip-4	0	1	0	0	0	0	0	0
Lip-5	0.2	0	0	0	0	0	0,8	0
Lip-6	0.25	0	0	0	0.5	0	0.25	0
Lip-7	0.25	0	0	0	0.5	0.25	0	0
Lip-8	0.33	0	0	0	0	0	0.33	0.33
Lip-9	0.5	0	0	0	0.5	0	0	0
Lip-10	0.5	0.5	0	0	0	0	0	0

**Table 2:** Polydispersity index (PDI) of bare liposomal formulations and after 1-h incubation with HP.

Samples	PDI	
	−HP	+HP
Lip-1	0.14 ± 0.04	0.12 ± 0.04
Lip-2	0.11 ± 0.01	0.17 ± 0.05
Lip-3	0.08 ± 0.01	0.13 ± 0.01
Lip-4	0.12 ± 0.02	0.14 ± 0.01
Lip-5	0.10 ± 0.02	0.16 ± 0.05
Lip-6	0.10 ± 0.02	0.17 ± 0.01
Lip-7	0.12 ± 0.02	0.20 ± 0.04
Lip-8	0.11 ± 0.02	0.18 ± 0.03
Lip-9	0.15 ± 0.01	0.22 ± 0.02
Lip-10	0.14 ± 0.01	0.20 ± 0.01

**Table 3:** Micrograms of proteins bound to liposomal formulations after 1-h incubation with HP.

Samples	Protein (μg/μL)
	+HP
Lip-1	4.9 ± 0.4
Lip-2	5.3 ± 0.6
Lip-3	4.8 ± 0.5
Lip-4	9.0 ± 0.9
Lip-5	3.1 ± 0.4
Lip-6	4.6 ± 0.3
Lip-7	4.1 ± 0.4
Lip-8	3.7 ± 0.4
Lip-9	9.5 ± 0.9
Lip-10	6.6 ± 0.5

**Table 4:** Number of proteins adsorbed on liposomal formulations after 1-h incubation with HP.

Samples	#Identified Proteins
Lip-1	220
Lip-2	140
Lip-3	205
Lip-4	170
Lip-5	174
Lip-6	202
Lip-7	206
Lip-8	180
Lip-9	222
Lip-10	190
